# Landfill site suitability analysis for sustainable solid waste management using AHP and GIS in Burdur Lake Basin, Türkiye

**DOI:** 10.1016/j.mex.2025.103555

**Published:** 2025-08-05

**Authors:** İbrahim İskender Soyaslan

**Affiliations:** Burdur Mehmet Akif Ersoy University, Faculty of Engineering and Architecture, Department of Civil Engineering, 15030 Burdur, Türkiye

**Keywords:** Waste management, Landfill, Suitablility analysis, AHP

## Abstract

A critical challenge confronting cities today is the disposal or storage of generated waste. Municipal solid waste landfill site appraisal is essential for sustainable and ecologically responsible solid waste management. In this study, a suitability analysis for municipal solid waste disposal sites in the Burdur Lake Basin was conducted using multi-criteria decision-making tools, specifically the Analytic Hierarchy Process and Geographic Information System. AHP established a hierarchy structure that facilitated the determination of the significance levels of the parameter and sub-parameter through the assignment of weights. A landfill suitability map was created using these parameter weights in a GIS analysis. The study region is categorized into four landfill suitability classes: inappropriate (35.72 %), low suitability (23.75 %), suitable (18.14 %) and high suitability (22.38 %). Thus, it has been determined that the conditions of the existing landfill site in Burdur province are met, and two alternative landfill sites have been identified for future use.

The study offers valuable insights for decision-makers engaged in sustainable solid waste management in Türkiye and comparable regions.

– How to find an environmental landfill to achieve sustainable development

- What regional characteristics and parameters are considered in the selection of the landfill site.

## Graphical abstract

**Figure:**
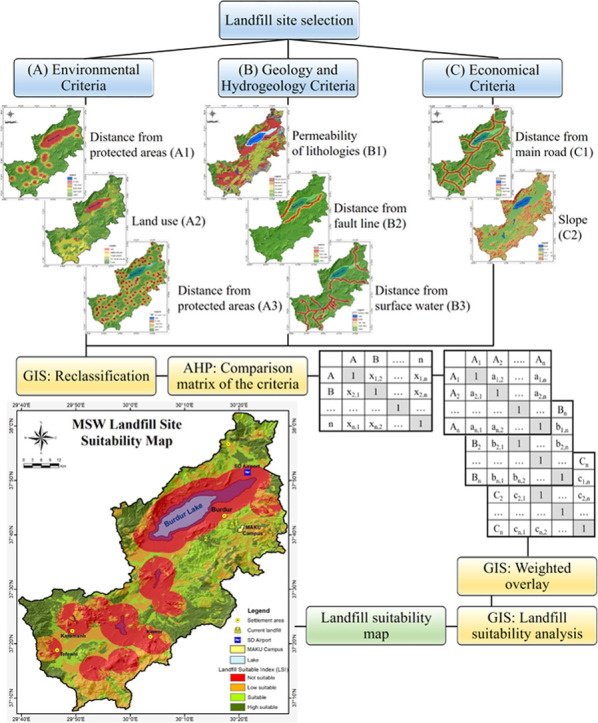

## Specifications table

**Table:** 

Goal	Main criteria	Criteria
MSW Landfill suitability analysis	(A). Environmental criteria	(A1) Distance from protect areas
		(A2) Land use
		(A3) Distance from settlement areas
	(B). Geology and hydrogeology criteria	(B1) Permeability of lithological units
		(B2) Distance from fault line
		(B3) Distance from surface water
	(C). Economic criteria	(C1) Distance from main road
		(C2) Slope

Subject area	Earth and Planetary Sciences	
More specific subject area	Geology, hydrogeology, hydrology, GIS	
Name of your method	MCDC: multi-criteria decision making	
Name and reference of original method	AHP: Analytic Hierarchy ProcessSaaty TL (1980) The analytic hierarchy process. McGraw Hill, New York.	
Resource availability	*None*	

## Background

As cities grow, considerable amounts of residential and industrial rubbish must be properly disposed of or stored [1,2]. Emerging economies with rapid urbanization have solid waste concerns [3,4]. Municipal solid waste (MSW) landfill suitability research focuses on emerging nations such Africa, the Middle East, the Far East, and Turkey [[5], [6], [7], [8], [9]]. Population growth, shifting consumer tastes, and rapid technological advancements have all contributed to an increase in trash [[10], [11]]. Despite the risks to the environment, waste disposal is the most cost-effective. This rubbish management strategy is preferred by both developing and developed countries [[12], [13], [14]]. Waste management can produce dust, odors, gas, and smoke [15]. Storing unburned coal, wood, and other materials can lead to winter fires. Residential neighborhoods near MSW landfills may experience significant environmental and health risks [16,17]. These characteristics must be considered in landfill suitability assessments.

After deciding on a waste storage technique, solid waste storage is the most important consideration. Many national and international laws assess MSW landfill suitability [[18], [19], [20], [21]]. A location that is safe for both water and the environment is required for public health [22,23]. MSW landfills alter land usage and ecosystems. Local governments rely on economic suitability assessments without a strategy [[24], [25], [26]]. This hurts the environment and the economy, lowering local standards of living [27]. Choose the finest site based on acceptable criteria, not restrictive ones. Researchers anticipate that the proposed storage location will have minimal environmental and public health implications [28,29]. However, MSW landfills are technical structures that, if not properly constructed, can cause irreversible environmental damage. We must employ cutting-edge computer technology and procedures to assess the site's environmental impact. The simplest technique to select the best storage location is to categorize components by working area characteristics and rank them based on site suitability study impact. This study incorporated anthropogenic, natural, social, environmental, geological, hydrogeological, meteorological, and economic variables [9,30,31]. Regardless of the technique, application area aspects influence selection.

An MSW landfill suitability study necessitates substantial environmental and geographical data gathering, GIS processing, and analysis [32]. This strategy focuses on using GIS analytical and mathematical methods to incorporate digital data into decision-making. This research contends that multi-criteria decision-making (MCDM) approaches based on the analytic hierarchy process (AHP) and GIS are fair and objective landfill site selection methods [33,34]. Recent MSW landfill suitability studies employing this method have reached this conclusion [13,35].

MCDM and GIS can easily solve landfill suitability investigations [[36], [37], [38], [39]]. Remote sensing using AHP and GIS for MCDM analysis is gaining popularity [[40], [41], [42], [43], [44], [45]]. AHP is used in complex geographical analysis with contradictory MCDM criteria [[46], [47], [48]]. Some scholars have combined fuzzy, MCDM, and AHP [[49], [50], [51]]. Recent developments in multi-criteria decision analysis and GIS have addressed technological challenges [25,52]. A global study investigated MSW landfill feasibility using GIS-based MCDM [31,53,54].

## Method details

GIS is a system that collects, stores, analyzes, and graphically visualizes spatial data related to attribute information, thereby creating themed maps. This system is highly appropriate for the storage, analysis, and transformation of criteria for landfill suitability studies into thematic maps. In recent years, GIS have emerged as a crucial instrument for addressing environmental concerns and doing land use studies. In a GIS context, analyzing spatially represented criteria correlations, integrating various information sources, and visually presenting the study results is simple and straightforward. The utilization of GIS technology and thematic digital maps enables the identification and analysis of both natural and artificial criteria within the study region.

It would be inaccurate to choose a subset of criteria from the appropriateness analysis and assert that these are the appropriate criteria. The criteria may differ based on the distinctive characteristics of the study region. Neglecting seismicity in earthquake-prone regions with active fault lines may result in significant inaccuracies. It is fundamentally mistaken to disregard drainage areas or proximity to rivers as factors in regions characterized by heavy rainfall and surface runoff. Moreover, we must apply factors such as geology, slope, protected zones, proximity to major roads, settlement area and land utilization, regardless of regional attributes. These examples show that in suitability analysis studies, we must assess all criteria and choose the most pertinent ones based on the distinctive characteristics of the study region. Moreover, the criteria selection technique may result in significant inconsistencies and inaccuracies in the study's outcomes. An active MSW landfill facility is located approximately 10 km southwest of the Burdur province settlement area. The suitability analysis of the existing MSW landfill site in the study area has been conducted, and potential locations for a new MSW landfill site area for future use have been identified.

Eight factors have been designated for the appropriateness examination of the municipal solid waste site. The parameters have been digitized and stored in a Geodatabase established with ArcGIS 10.1 software within a GIS environment. Thereafter, the vector data were transformed into raster data, and all parameters were georeferenced in accordance with the Geographic Coordinate System. A continuous surface for the parameters was generated in grid regions via the IDW method, with a cell size of 25 × 25. The National Turkish Solid Waste Control Regulation has established sub-criteria and buffer levels for all metrics. Buffering procedures were conducted for five parameters utilizing these buffer values. Upon completion of the buffering process, the reclassification was finalized, and the sub-criteria pertaining to the pre-AHP criteria were established. In the implementation phase, all users are permitted to establish and utilize their own standard and buffer zones in accordance with national or international regulations.

In the AHP stage, each criteria is ordered according to the flow equation, and a weighting factor is applied to each criterion. The weighting variables are finally utilized in the weighted overlay function inside ArcGIS 10.1. Weights and scores are allocated to each criteria and sub-criterion. Finally, a weighted overlay analysis is done in ArcGIS 10.1 to create the MSW landfill site suitability map.

### Geo-environmental setting of the study area

**Fig. 1:**
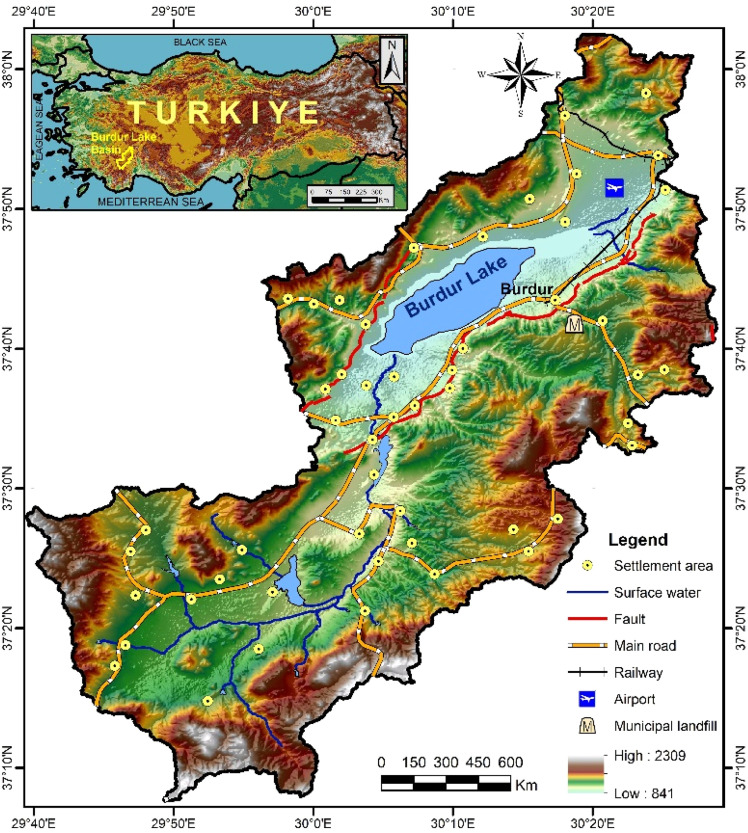
Map of the study area (Burdur Lake Basin in Türkiye).

The Burdur Lake Basin (BLB) is situated in the western section of the 'Isparta Bend' fault line and the north-eastern segment of the Burdur-Fethiye fault line [55]. The BLB includes an area of 3152 km², predominantly spanning the provincial boundaries of Burdur, Isparta, and Antalya. The study area is situated between 29°40‘−30°30′ East longitude and 37°05‘−38°05′ North latitude, including an approximate area of 1824 km², which includes Lake Burdur (Fig. 1).

Faults extending from the southwest to the northeast delineate a tectonic depression basin that defines BLB. In the northern and southern regions, elevated hills provide a horst structure, whereas the central low-lying plain of the basin constitutes a graben. The study region is hence designated as the Burdur Graben. The graben situates Lake Burdur at its lowest altitude. A significant concern in the basin is the dramatic decrease in the water level of Lake Burdur over the last fifty years [56,57]. The primary causes of this occurrence are the construction of ponds on the rivers that supply the lake, uncontrolled groundwater abstraction, and global climate change. Due to its status as an enclosed basin, Lake Burdur's water is saline.

Consequently, it is unsuitable as a source of potable water. Due to its seasonal hosting of almost one hundred migrating bird species, it receives protection. The Ramsar Convention designated Lake Burdur and its vicinity as a "Wildlife Protection Area" in 1993, followed by a "Ramsar Convention Protected Area" in 1994, and a first-degree "archaeological protected area" in 1998. The purpose of these limits is to protect the unique ecological, environmental, and historical significance of Lake Burdur and its surroundings.

### Definition of criteria and sub-criteria

The MSW landfill suitability investigation procedure requires the evaluation of numerous natural and artificial criteria. The factors for selecting an appropriate and precise landfill location are more significant than the methods applied. The chosen criteria, informed by the distinctive attributes of the research region and its surrounding areas, directly influence the site selection process. The objective is to determine the appropriate criteria for the study region situated in southwestern Türkiye. The objective is to determine the criteria used in similar investigations carried out globally. The parameters used in landfill suitability studies carried out at various times across diverse global locations have been evaluated (Table 1).

**Table 1:** Criteria used in methods in references.

References*	1	2	3	4	5	6	7	8	9	10	11	12	13	14
Country	Jordan	S. Arabia	Tanzania	Türkiye	Oman	Türkiye	Brazil	Iran	Iraq	Turkiye	Sri Lanka	Ethiopia	India	Pakistan
Geology			+	+		+	+			+		+	+	
Hydrogeology		+		+	+	+		+	+					+
Surface water	+	+	+	+		+	+	+	+	+	+	+	+	+
Water channels			+											
Soil type	+	+	+				+	+	+		+	+	+	+
Fault			+			+	+			+				
Earthquake epicentres			+				+							
Population					+						+			
Elevation		+	+	+	+				+		+			
Slope	+	+	+	+	+	+	+	+	+	+		+	+	+
Settlement Area	+	+	+		+	+		+	+	+	+			+
Land use	+	+		+	+	+		+	+	+	+	+	+	
Road	+	+	+	+	+	+		+	+	+		+	+	+
Railway			+						+					+
Boreholes			+		+		+							
Airport			+		+	+								
Vegetation					+									
Protect areas			+	+		+	+		+					+
Sensitive sites			+			+								
Waste generation place								+			+			
Precipitation						+	+				+			
Temperature						+	+							

⁎ 1: [39], 2: [40], 3: [4], 4: [51], 5: [27], 6: [9], 7: [58], 8: [54], 9: [13], 10: [59], 11: [60], 12: [20], 13: [61], 14: [26].

In the analysis of fourteen distinct research studies, one study used criteria such as caves, water quality, drainage density, valleys, vegetation, pipelines, power lines, sensitive ecosystems, and erosion. Table 1 presents the criteria used in a minimum of two research studies. The obtained criteria indicate the possibility of incorporating additional criteria, contingent on the study area's characteristics. This study included eight criteria: distance from protected areas, land use, distance from settlement areas, Permeability of lithological units , distance from fault line, distance from surface water, distance from main road and slope. The eight criteria have been categorized into three distinct primary criteria for independent evaluation (Table 2).

**Table 2:** Main Criteria and criteria used in MSW landfill suitability analysis.

Goal	Main criteria	Criteria
MSW Landfill suitability analysis	(A). Environmental criteria	(A1) Distance from protect areas
		(A2) Land use
		(A3) Distance from settlement areas
	(B). Geology and hydrogeology criteria	(B1) Permeability of lithological units
		(B2) Distance from fault line
		(B3) Distance from surface water
	(C). Economic criteria	(C1) Distance from main road
		(C2) Slope

Because of its proximity to Lake Burdur, which is located within protected zones, the airport in the BLB was not considered a distinct criterion. Furthermore, it has been assessed under the settlement area sub-criterion of the airport land use criterion. The railway track within the basin goes parallel to the Burdur highway once more. The application of the distance to the main road criterion precluded the use of the railway criterion.

**Fig. 2:**
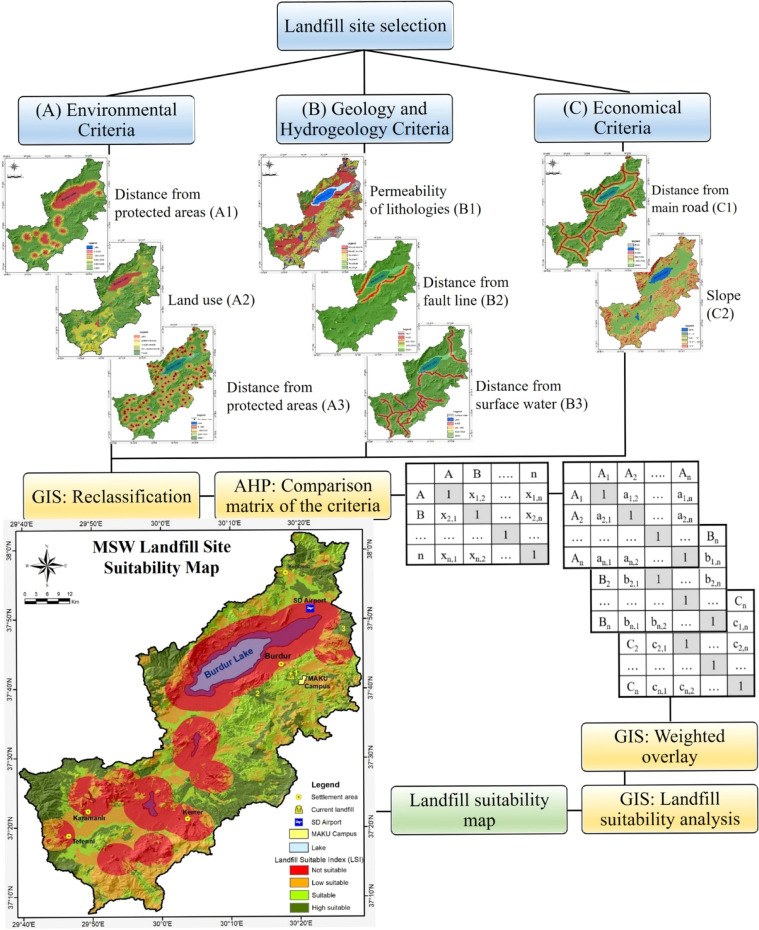
The flowchart of the used methodology.

This study employed GIS and AHP approaches collaboratively for the selection process of MSW landfill sites. The methodology has four stages: (a) elimination of inappropriate locations, (b) identification of evaluation criteria, (c) creation of standard maps, and (d) development of a suitability index and land suitability map utilizing MCDM. The study identifies eight criteria categorized into three primary groups: environmental, geological-hydrogeological, and economic variables (Fig. 2). Turkish regulations and foreign literature have guided the selection of these parameters. Although environmental criteria were assessed with greater sensitivity, economic criteria became more significant due to financial limitations and land costs. The geological-hydrogeological criteria emphasize the geological structure of the region, surface waters, groundwater, and the preservation of natural resources. During the evaluation procedure, each criterion was assessed using a score system from 0 to 10. Turkish rules and international literature have validated the analysis results. This process provides a holistic approach that considers environmental, geological-hydrogeological, and economic sustainability.

### Method limitations

Conventional techniques often exhibit several constraints, including their simplicity and subjectivity in identifying appropriate landfill sites. These strategies often rely on fundamental characteristics, such proximity to metropolitan regions or land expenses. This circumstance hinders a comprehensive investigation of several aspects and may result in inferior site decisions. Moreover, these conventional techniques may insufficiently account for environmental issues, potentially resulting in the selection of places that might adversely affect ecosystems, water quality, or air quality. Conventional techniques may inadequately account for the equilibrium among economic, environmental, geological-hydrogeological, and economic factors. This complicates the ability to make deliberate and lasting judgments that reconcile many variables. The use of MCDM is essential to address these constraints. MCDM approaches provide a more thorough and objective decision-making process by integrating several elements. Parameter selection aids in discovering and selecting suitable parameter groupings, such as environmental, geological-hydrogeological, and economic factors. The allocation of weights signifies the relative significance of the criteria, enabling equilibrium and averting the predominance of any singular criterion in the decision-making process.

In addition to these positive features, MCDM methods also have serious limitations in the selection of suitable solid waste disposal sites. The presence of numerous alternatives in the classification, ranking, and selection processes of the parameters to be used makes the application of the method challenging. To eliminate this difficulty, experts who are knowledgeable about the regional characteristics are needed. After the parameters are selected, the inability to compare these parameters with each other is one of the significant limitations of the method. The sum of the parameter weights being 1 and this weighting being determined by the decision-maker sometimes leads to criticisms. These parameter weights, determined according to the characteristics of the area where the solid waste storage site is located, can vary in each application. This situation is sometimes considered a limitation of the method and leads to criticism.

These limitations can be overcome by obtaining expert opinions with regional characteristics during the decision-making process and parameter selection. However, it is not possible to produce a single parameter and weighting template that can be applied worldwide. The parameters and weights to be used in the selection of suitable solid waste disposal sites vary according to regional characteristics.

### Analytical hierarchy process (AHP) principle

**Fig. 3:**
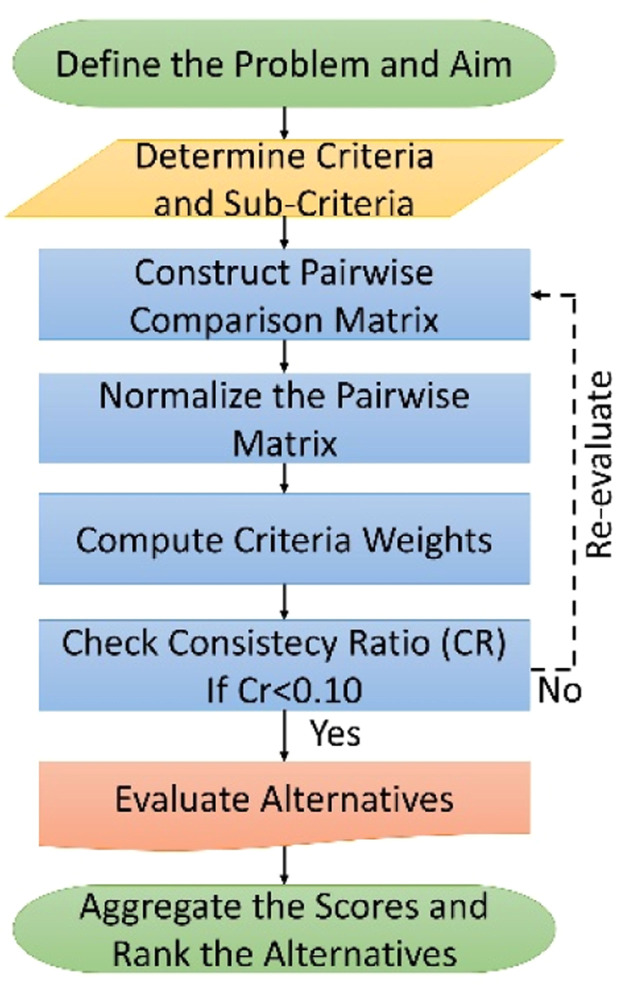
AHP flowchart.

One of the most preferred methods in MSW landfill suitability analysis studies is the multiple-criteria decision-making (MCDM) analysis. MSW landfill suitability analysis is defined as a complex process that involves MCDM analysis. This study employed the AHP method for the MCDM analysis (Fig. 3). The AHP method utilizes pairwise comparison to establish priority criteria. An absolute evaluation scale, as used in parameter comparisons, illustrates the degree of superiority of the parameters over each other [39,62,63]. The pairwise comparisons of the parameters assign a coefficient to each criterion, signifying its importance. The weights of the coefficients for all parameters are determined so that their total equals one hundred. The region's geology, topography, hydrology, hydrogeology, and land use characteristics have determined the parameter weights. To evaluate the dual importance of parameters, primary coefficients ranging from 2 to 9 for very important and from 1/2 to 1/9 for less important are used (Table 3). For the case where two parameters have equal importance, a coefficient of 1 is used.

**Table 3:** AHP evaluation scale [60].

Definition	Coefficient	Explanation
More Important	9	Extremely
	7	Very strogly
	5	Strongly
	3	Moderately
Equally	1	Equally
Less important	1/3	Moderately
	1/5	Strongly
	1/7	Very strongly
	1/9	Extremely

These coefficients indicate the degree of superiority of one parameter over another (2–9), the extent of inferiority (1/2–1/9), or equality between two parameters (1). The crucial stage in the application of AHP is defining the coefficients and constructing the NxN comparison matrix derived from these coefficients. (Eq. (1)).

(1) $$\begin{array}{cc} \left[\begin{array}{cccc} a_{11} & a_{12} & \ldots & a_{1N}\\ a_{21} & a_{22} & \ldots & a_{2N}\\ \cdot & \cdot & \ldots & \cdot \\ \cdot & \cdot & \ldots & \cdot \\ \cdot & \cdot & \ldots & \cdot \\ a_{N1} & a_{N2} & \ldots & a_{NN} \end{array}\right]={\left[a_{ij}\right]}_{NxN} & i,j=1,2....N \end{array}$$

In the equation, N is the number of parameters, i and j are parameters, and aij is the degree of preference of parameter i over parameter j. In this comparison matrix, the value on the diagonal of the matrix, where the parameters are compared with themselves, will be "1." For the parts below the diagonal value, the formula in Eq. (2) is used [62,64].

(2) $$a_{i,j}=\frac{1}{a_{j,i}}$$

The consistency of the values in the comparison matrix needs to be tested. For this purpose, the Consistency Index (CI) is calculated (Eq. (3)):

(3) $$CI=\frac{\left(\lambda_{maz}-n\right)}{\left(n-1\right)}$$

In Eq. (1), $\lambda_{\max}$ is the basic decision matrix value (maximum eigenvalue) and n is the number of parameters used. The maximum eigenvalue $\lambda_{\max}$ is calculated using Eq. (4).

(4) $$\lambda_{\max}=\frac{\sum_{i=d}^{n}d_{i}}{n}$$

The concluding stage of the AHP study involves computing the Consistency Ratio (CR), which assesses the consistency of the evaluations relative to a large sample of random judgments. Subsequent to computing the CI value, the "Consistency Ratio" (CR) is determined (Eq. (5)).

(5) $$CR=\frac{CI}{RI}$$

In the CR equation, CI indicates the consistency index, whereas RI represents the random index. RI varies based on the quantity of parameters compared (Table 4). In this investigation, the RI value was determined to be 1.41, as nine criteria (*n* = 8) were used. The CR value ranges from 0 to 1, and a value below 0.1 (10 %) indicates that the matrix is consistent [65]. If the result is significant, we modify the superiority values of the parameters to stay below 0.1 [31].

**Table 4:** Number of parameters and RI values.

N	1	2	3	4	5	6	7	8	*9*	10
RI	0	0	0.58	0.90	1.12	1.24	1.32	1.41	*1.45*	1.49

In the CR equation, CI refers to the consistency index and RI refers to the random index. RI varies based on the quantity of the parameters under comparison. Table 4. This study determined the RI value at 1.41 by using eight criteria (*n* = 8). The CR value ranges from 0 to 1, and a value below 0.1 (10 %) indicates that the matrix is consistent [61]. For higher values, the superiority parameters are modified to remain below 0.1 [31].

### Geographic information system (GIS)

The parameters used in the MSW landfill suitability analysis were sourced from official institutions, field studies and measurements and subsequently integrated into the GIS environment. Groundwater depth measurements were occurred in the study area between May and October 2023. The data was transformed into a static level, incorporated into the GIS environment, and interpolated utilizing the IDW method. Long-term average rainfall data for the study area was sourced from the General Directorate of State Meteorological Affairs. Precipitation data were imported into a GIS environment and interpolated via the IDW method to generate an average precipitation distribution map for the study area. The digital elevation model (DEM) for BLB was derived from the digitization of topographic maps at a scale of 1:25,000. The geological map of the study area was sourced from the General Directorate of Mineral Research and Exploration and subsequently refined through field studies for its intended application. A hydrogeology map was developed by analyzing the hydrogeological properties of the lithological units depicted in the geological map. The land use map was developed in a GIS environment utilizing 1:100,000 scale digital data as part of the CORINE project (2018). ArcGIS 10.1 software transformed all parameter maps into a grid map with a resolution of 25 × 25 m within a GIS environment. All maps utilized and generated inside the GIS environment employ the Geographic Coordinate System (degrees, minutes, seconds).

Geographical interpolation, geographical querying, and spatial data visualization are essential GIS capabilities utilized in spatial decision-making processes. The spatial interpolation procedure can estimate unknown values of specific elements. The techniques employed for spatial interpolation include inverse distance weighting (IDW), ordinary kriging (OK), and spline interpolation. The IDW and OK methods are the most often utilized, compared, and endorsed interpolation approaches. This study employed the IDW method as the interpolation approach.

### Inverse distance weighting (IDW) interpolation method

The interpolation approach involves ascertaining unknown point values by computing them from known point values. Values of unknown points are computed using the attribute table values of known points to generate a continuous surface. The IDW interpolation method operates on the premise that proximate values exhibit greater similarity than those that are further distant. The values nearest to the place being computed exert a stronger effect on the resultant value than those that are farther away. Consequently, IDW posits that the measured point exerts a distance-decreasing influence. It assigns greater significance to points in proximity to the target point than to those at a greater distance.

IDW conducts impact analysis by applying distance-based weighting. In this approach, an increase in distance diminishes the weight value. Eq. (6) presents the equation that the IDW approach employs.

(6) $$\hat{Z}\left(s_{0}\right)=\left[\sum_{i=1}^{n}w\left(d_{i}\right)Z\left(s_{i}\right)\right]/\sum_{i=1}^{n}w\left(d_{i}\right)$$

In the Eq. (6) $\hat{Z}\left(s_{0}\right)$, Z(si) represent the estimated and observable values of s_0_ and s_i_ at their respective locations; "n" is the number of measured sample points used in the estimation; "w" is the weight function; and d_i_ represents the distance between the values s_0_ and s_i_. The weighting function can significantly influence the interpolation results, depending on the nature of the IDW expression.

The Spatial Analyst Tools-Interpolation-IDW command in Arc Toolbox of ArcGIS 10.1 has been used in this method's implementation. Upon executing this command, the parameter must be chosen in the "Input point features" section of the IDW menu that appears, the value for interpolation should be specified in the "z value field" section, and the destination for the output raster file must be designated in the "Output raster" section. Furthermore, under the "Environments" section, inside the "Processing Extend" part, the "Extent" menu is accessed, and the watershed border for interpolation is chosen in the "Same as layer" section. The watershed boundary is ultimately chosen using the "Mask" subcommand under the "Raster Analysis" section. Consequently, our newly interpolated raster data, generated by the application, is ready.

The IDW interpolation method is a deterministic technique that estimates values at unknown spatial locations by weighting them based on their distances from known data points. This strategy is founded on the idea of spatial autocorrelation, which posits that proximate sites exert a stronger influence. In the IDW technique, the influence of each known location on the prediction is determined by applying a weight based on the inverse of the distance to that point raised to a specific exponent, referred to as the "power parameter" (denoted as ρ). The power parameter is a crucial variable that influences the sensitivity and texture of the interpolation surface. When the ρ-value is maintained at a low level (for instance, 1), the influence of remote points amplifies, leading to a more uniform and generalized surface. Conversely, as the ρ value escalates (for instance, 2 or greater), the impact of proximate points prevails, resulting in a more localized, intricate, although also more irregular interpolation surface. This circumstance renders elevated ρ values more efficacious in emphasizing abrupt alterations, particularly in datasets characterized by diverse distribution. The right power parameter must be meticulously selected based on data density, distribution, and the analytical objective; failure to do so may lead to biased or distorted spatial surfaces from interpolation. Consequently, the efficacy of the IDW approach predominantly hinges on the accurate optimization of the power parameter.

### MSW landfill suitability analysis criteria

Three primary criteria have been established for the selection of the MSW Landfill Site: environmental (A), geology-hydrogeology (B) and economic (C). The environmental protection strategy aligns with the allocation of the greatest weight to environmental issues and the least weight to economic concerns. The weight numbers indicate the significance of the primary criterion in the process. The methodology employed three distinct weighing techniques to classify the primary criterion, criteria, and sub-criteria, thereby facilitating a more accurate assessment of the criteria.

### Distance from protected areas (A1)

**Fig. 4:**
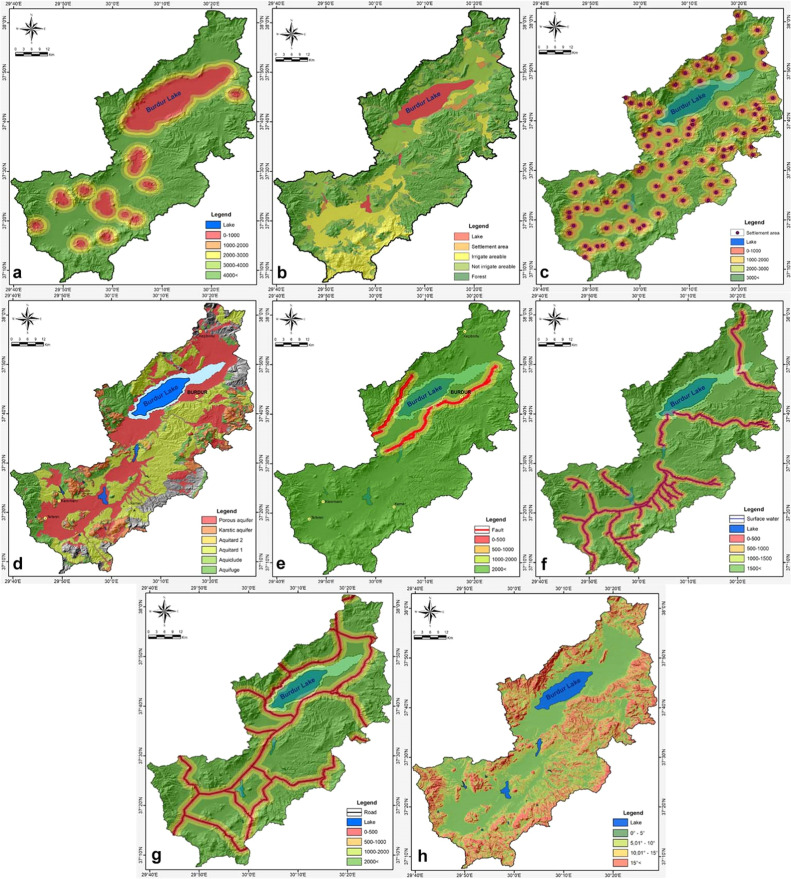
(a) Distance from protected areas. (b) Land use. (c) Distance from settlement areas.(d) Permeability of lithological units. (e) Distance from fault line. (f) Distance from surface water. (g) Distance from main road. (h) Slope.

The study area includes protected regions, specifically Lake Burdur and nearby dam lakes protected by the Ramsar Convention. Lake Burdur accommodates several migrating bird species. On the other hand, dam lakes serve both fishing and agricultural irrigation purposes. The lake reservoir regions represent the most significant conservation zones within the research area, based on their utilization. The protected areas have been evaluated utilizing IDW inside a GIS framework. As a result, we established five distinct buffer zones: 0–1000, 1000–2000, 2000–3000, 3000–4000 and above 4000 m (Fig. 4a).

### Land use (A2)

MSW dump locations select land usage as an environmental criterion. Land use encompasses a limited subset of criteria, including lakes, settlement areas, and irrigated arable land, for the examination of landfill appropriateness. The land use data is derived from the CORINE (Coordination of Information on the Environment) land cover map, utilizing the CLC2018 dataset at a 1:100,000 scale for countries within the European Union (EU). Based on its intended use, we have categorized the land use data into five sub-criteria: lake, settlement area, irrigable arable land, non-irrigable arable land, and forest (Fig. 4b).

### Distance from settlement areas (A3)

Due to environmental sensitivity, the dump site must be located at a considerable distance from populated areas. According to the Regulation on the Controlled Storage of Wastes in Turkey, residential zones must be at least 1000 m away from solid waste storage facilities [66]. The distance from settlement areas was studied in a GIS context utilizing inverse distance weighting (IDW). This analysis assessed distance values from settlement areas based on four sub-criteria: 0–1000 m, 1000–2000 m, 2000–3000 m, and over 3000 m (Fig. 4c). The assessment includes all urban regions, including cities, districts, and village settlements.

### Permeability of lithological units (B1)

Based on field studies and a literature review in the subject area, we have produced a geological map at a scale of 1:25,000. ArcGIS software has digitized the map, transforming it into a grid map. The geological units in the basin have been categorized into ten categories according to their permeability characteristics, corresponding with the study's aims. Based on the hydrogeological characteristics of these lithological units, six criteria have been formulated according to their permeability qualities. The units have been classified according to their aquifer capacities from lowest to highest as aquifuge, aquilude, aquitard1, aquitard2, karstic aquifer, and porous aquifer (Fig. 4d).

### Distance from fault line (B2)

The first-degree earthquake zone, the most seismically hazardous region, is where the research area is located. Historically, two significant earthquakes occurred in 1914 (magnitude 7.1) and 1971 (magnitude 6.2) along the fault lines within the study area, resulting in casualties and property damage. Therefore, we have selected landfill sites based on the criterion of distance from active fault lines. The distance from the fault line criterion was examined in the GIS context utilizing inverse distance weighting (IDW). The study identified four distinct sub-criteria for distance from the fault line: 0–500 m, 500–1000 m, 1000–2000 m, and over 2000 m (Fig. 4e). Buffer zones have been established in a GIS context utilizing these sub-criteria distances for active fault lines.

### Distance from surface water (B3)

The Turkish Water Pollution Control Regulation restricts the establishment of waste disposal sites in proximity to surface water bodies. Therefore, we established four distinct buffer zones surrounding all surface waters at 500-meter intervals using inverse distance weighting (IDW). The first buffer zone spans 0–500 m, the second buffer zone extends from 500–1000 m, the third buffer zone ranges from 1000–1500 m, and the final buffer zone encompasses areas beyond 1500 m (Fig. 4f).

### Distance from main road (C1)

The distance from the main road has been established as a sub-criterion, considering the transportation costs associated with transporting solid waste to the disposal site. In the GIS context, sub-criteria were established utilizing inverse distance weighting (IDW) for distance ranges of 0–500 m, 500–1000 m, 1000–2000 m, and over 2000 m (Fig. 4*g*). The nearest buffer zone possesses the greatest weight, while the farthest buffer zone carries the least weight.

### Slope (C2)

The slope criterion determines the direction of surface runoff, flow rate, mass movements, and the possible distribution of solid waste within the landfill during its operational phase. Therefore, the landfill site prefers locations with a reduced slope gradient. Slope values were assessed utilizing digital elevation model (DEM) data within the GIS environment. The analysis evaluated the slope criterion across four sub-criteria: 0°−5°, 5°−10°, 10°−15°, and above 15° (Fig. 4h).

## Method validation

An examination of MSW landfill appropriateness has been performed utilizing three distinct methodologies. The primary criteria in the initial phase were assigned weights of 53.96 % for environmental criteria, 29.70 % for geological and hydrogeological criteria, and 16.34 % for economic factors. The second procedure assigned weights to the criteria that fell under the three primary categories. The criteria include distance from protected area (58.15 %), land use (30.90 %), distance from settlement areas (10.95 %), permeability of lithological units (53.96 %), distance from fault line (29.70 %), distance from surface water (16.34 %), distance from main road (33.33 %), and slope (66.67 %). The sub-criteria have been categorized according to the attributes of the research region and assigned weights based on their impact on the MSC landfill suitability assessment (Table 5).

**Table 5:** Criteria weights obtained by AHP to be used in landfill suitability analysis.

PROCESS 1			PROCESS 2			PROCESS 3			
MAIN CRITERIA	W*	CR⁎⁎	CRITERIA	W*	CR⁎⁎	SUB-CRITERIA	W*	CR⁎⁎	Σ W*
ENVIRONMENTAL CRITERIA	0.5396	0.0088	DISTANCE FROM PROTECTED AREAS (M)	0.5815	0.0035	0–1000	0,0413	0.0094	0.0130
						1000–2000	0.0910		0.0286
						2000–3000	0.1669		0.0524
						3000–4000	0.3166		0.0993
						4000<	0.3842		0.1206
			LAND USE	0.3090		LAKE	0.0471	0.0047	0.0079
						SETTLEMENT AREA	0.0471		0.0079
						IRRIGATE AREABLE	0.1455		0.0243
						NOT IRRIGATE AREABLE	0.2944		0.0491
						FOREST	0.4659		0.0777
			DISTANCE FROM SETTLEMENT AREAS (M)	0.1095		0–1000	0.0614	0.0062	0.0036
						1000–2000	0.1619		0.0096
						2000–3000	0.2723		0.0161
						3000<	0.5044		0.0298
GEOLOGY AND HYDROGEOLOGY CRITERIA	0.2970		PERMEABILITY OF LITHOLOGICAL UNITS	0.5396	0.0088	AQUIFUGE	0.4004	0.0066	0.0642
						AQUICLUDE	0.2680		0.0430
						AQUITARD1	0.1174		0.0188
						AQUITARD2	0.1044		0.0167
						KARSTIC AQUIFER	0.0565		0.0091
						POROUS AQUIFER	0.0533		0.0085
			DISTANCE FROM FAULT LINE (M)	0.2970		0–500	0.0758	0.0039	0.0067
						500–1000	0.1516		0.0134
						1000–2000	0.2827		0.0249
						>2000	0.4899		0.0432
			DISTANCE FROM SURFACE WATER (M)	0.1634		0–500	0.0602	0.0030	0.0029
						500–1000	0.1171		0.0057
						1000–1500	0.2242		0.0109
						>1500	0.5985		0.0290
ECONOMIC CRITERIA	0.1634		DISTANCE FROM MAIN ROADS (M)	0.3333	0.00001	0–500	0.5313	0.0030	0.0289
						500–1000	0.2723		0.0148
						1000–2000	0.1428		0.0078
						2000<	0.0536		0.0029
			SLOPE (SO)	0.6667		0–5	0.5313	0.0030	0.0579
						5–10	0.2723		0.0297
						10–15	0.1428		0.0156
						15<	0.0536		0.0058

⁎ Weight (W).

⁎⁎ Consistency ratio (CR).

Finally, every category were assessed independently utilizing the AHP. The criterion map was generated with the weighted values of each buffer zone presented in Table 5 through ArcGIS software.

**Fig. 5:**
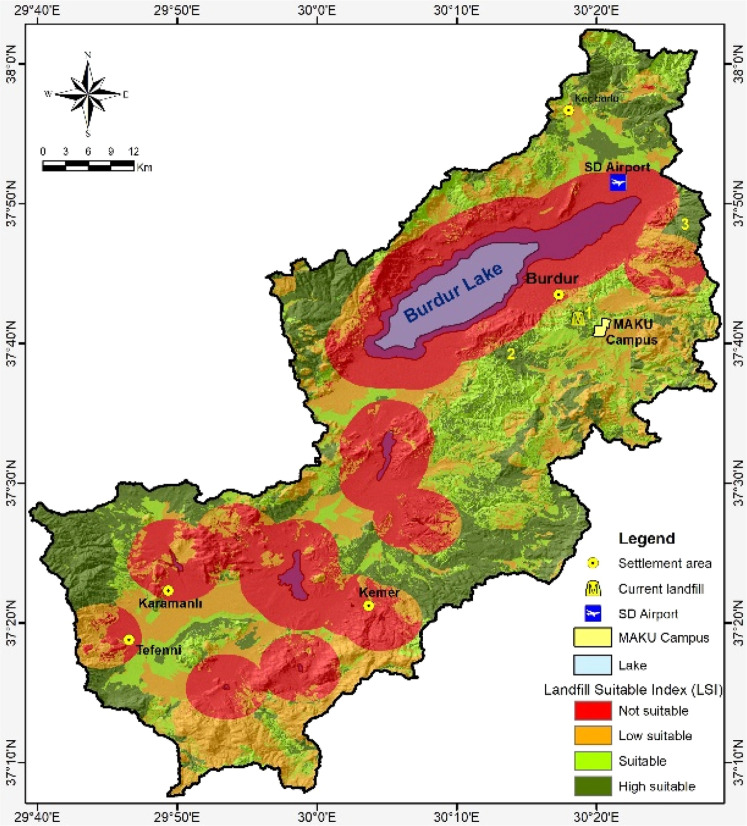
MSW landfill site suitability map for the Burdur Basin.

The weight values of the criteria and sub-criteria derived from AHP were transformed into GIS grid format. A suitability map was generated following the elimination of unsuitable restricted zones. The suitability map was generated via the GIS map calculator function and spatial analyst analysis. The equal interval method classified the study region into four distinct appropriateness categories. The suitability map delineates four distinct landfill suitability categories: unsuitable (35.72 %), low suitability (23.75 %), suitable (18.14 %), and high suitability (22.38 %) (Fig. 5).

The results of the AHP approach agree with the data obtained from field research. The Burdur Lake region, along with protected and restricted regions within the BLB, is considered unsuitable for MSW disposal in accordance with national regulations. The MSW landfill suitability investigation has revealed three distinct sites in proximity to Burdur province. Area 1 encompasses the presently utilized MSW landfil lsite in Burdur Province. Conversely, Areas 2 and 3 possess attributes that may function as viable alternatives to the current MSW landfill site in the forthcoming years. The additional appropriate places found through the investigation may serve as MSW landfill sites for other small communities. A comprehensive feasibility study should be conducted prior to utilizing the identified feasible sites throughout the entire basin. Feasibility studies are crucial for validating the findings of this research.

The selection of criteria is the most crucial phase in the appropriateness examination of MSW landfill sites. In establishing the criteria, it is essential to consider not only economic but also environmental and geological-hydrogeological factors. The criteria should consider the distinctive characteristics of the study area. The application of the criterion must consider both national and international regulations.

## Ethical statement

The work does not involve animal or human subjects. Additionally, data from social media platforms are not used in the work.

## CRediT authorship contribution statement

**İbrahim İskender Soyaslan:** Conceptualization, Methodology, Writing – original draft, Validation, Conceptualization, Software, Writing – review & editing.

## Declaration of competing interest

The author declares that there are no known competitive financial interests or personal relationships that could influence the work reported in this paper.

## Data Availability

The authors do not have permission to share data.
